# The Capsule Depolymerase Dpo48 Rescues *Galleria mellonella* and Mice From *Acinetobacter baumannii* Systemic Infections

**DOI:** 10.3389/fmicb.2019.00545

**Published:** 2019-03-18

**Authors:** Yannan Liu, Sharon Shui Yee Leung, Yatao Guo, Lili Zhao, Ning Jiang, Liyuan Mi, Puyuan Li, Can Wang, Yanhong Qin, Zhiqiang Mi, Changqing Bai, Zhancheng Gao

**Affiliations:** ^1^Department of Respiratory and Critical Care Medicine, Peking University People’s Hospital, Beijing, China; ^2^School of Pharmacy, The Chinese University of Hong Kong, Shatin, Hong Kong; ^3^State Key Laboratory of Pathogen and Biosecurity, Beijing Institute of Microbiology and Epidemiology, Beijing, China; ^4^Department of Respiratory and Critical Care Medicine, 307th Hospital of Chinese People’s Liberation Army, Beijing, China

**Keywords:** *Acinetobacter baumannii*, capsule depolymerase, *Galleria mellonella* model, mouse model, systemic infection, antivirulence

## Abstract

The emergence of multidrug- and extensively drug-resistant *Acinetobacter baumannii* has made it difficult to treat and control infections caused by this bacterium. Thus, alternatives to conventional antibiotics for management of severe *A. baumannii* infections is urgently needed. In our previous study, we found that a capsule depolymerase Dpo48 could strip bacterial capsules, and the non-capsuled *A. baumannii* were significantly decreased in the presence of serum complement *in vitro*. Here, we further explored its potential as a therapeutic agent for controlling systemic infections caused by extensively drug-resistant *A. baumannii.* Prior to mammalian studies, the anti-virulence efficacy of Dpo48 was first tested in a *Galleria mellonella* infection model. Survival rate of Dpo48-pretreated bacteria or Dpo48 treatment group was significantly increased compared to the infective *G. mellonella* without treatment. Furthermore, the safety and therapeutic efficacy of Dpo48 to mice were evaluated. The mice treated with Dpo48 displayed normal serum levels of TBIL, AST, ALT, ALP, Cr, BUN and LDH, while no significant histopathology changes were observed in tissues of liver, spleen, lung, and kidney. Treatment with Dpo48 could rescue normal and immunocompromised mice from lethal peritoneal sepsis, with the bacterial counts in blood, liver, spleen, lung, and kidney significantly reduced by 1.4–3.3 log colony-forming units at 4 h posttreatment. Besides, the hemolysis and cytotoxicity assays showed that Dpo48 was non-homolytic to human red blood cells and non-toxic to human lung, liver and kidney cell lines. Overall, the present study demonstrated the promising potential of capsule depolymerases as therapeutic agents to prevent antibiotic-resistant *A. baumannii* infections.

## Introduction

*Acinetobacter baumannii* is an opportunistic pathogen that often causes nosocomial infections, with the highest prevalence noted in immunocompromised and debilitated patients confined to intensive care units (ICUs) (Elhosseiny and Attia, 2018). Common *A. baumannii* infections include soft tissue infections (particularly in burn wards), urinary tract infections, post-surgical endocarditis, pneumonia, meningitis, and sepsis (Garcia-Quintanilla et al., 2013; Wong et al., 2017). Comparatively, community-acquired infections caused by *A. baumannii* were rare but severe with a mortality rate reported as high as 64% (Dexter et al., 2015). Due to the extensive use of broad-spectrum antibiotics, especially carbapenems which are the most potent and reliable β-lactam antibiotics for the treatment of serious *A. baumannii* infections (Garnacho-Montero and Amaya-Villar, 2010), many *A. baumannii* strains isolated from patient specimens have become highly antibiotic resistant. Surveillance data in 2017 from China Antimicrobial Resistance Surveillance System (CARSS) indicated that about 66.7 and 69.3% of *A. baumannii* strains were resistant to imipenem and meropenem, respectively (Hu et al., 2018). Colistin has been increasingly used as the last-resort treatment against most carbapenem-resistant *A. baumannii*. However, parental administration of colistin can cause serious side effects including nephrotoxicity and neurotoxicity (Wong et al., 2017). Furthermore, the rapid emergence of colistin-resistant *A. baumannii* presents huge threats to public health that no effective antibiotics for infections caused by this problematic superbug. Hence, there is an urgent need for the development of novel strategies alternative to conventional antibiotics to combat severe *A. baumannii* infections.

In recent years, the safety and efficacy of lytic phages in combating infections caused by drug-resistant bacteria was demonstrated in numerous *in vitro* and *in vivo* studies (Wright et al., 2009; Fish et al., 2016; Leitner et al., 2017; Schooley et al., 2017). However, the use of intact phages to treat bacterial infections may not be ideal because a phage as a complete virion is hard to standardize for mass production and preservation. Moreover, whole phage genomes may contain some genes of unknown function, making it difficult to predict the long-term safety of phage therapy. Therefore, increasing attention has been drawn to investigate the potential of phage proteins in controlling bacterial infections. The effectiveness of endolysins against gram-positive bacteria have been demonstrated *in vitro* and *in vivo* (Chang et al., 2017a,b; Cheng et al., 2017; Zhou et al., 2017). However, reports of endolysins against gram-negative-bacterial infections are relatively limited due to the thick outer membrane surrounding the cell wall, protecting the gram-negative bacteria from the peptidoglycan-degrading enzymes. Outer-membrane permeabilizers were generally required to elevate antibacterial activity of some endolysins (Oliveira et al., 2014, 2016; Dong et al., 2015).

Recently, phage-encoded polysaccharide depolymerases have shown great potential as antivirulent agents in treating gram-negative-bacterial infections *in vitro* and *in vivo* (Lin et al., 2014, 2017; Lai et al., 2016; Majkowska-Skrobek et al., 2016; Guo et al., 2017; Hsieh et al., 2017). They can degrade the bacterial surface polysaccharides to expose the interior of bacteria for host immune attack (Lin et al., 2014, 2017; Hsieh et al., 2017). Although the structure, genomic and biochemical characterization of *A. baumannii* depolymerases have been reported (Lai et al., 2016; Lee et al., 2017; Oliveira et al., 2017, 2018; Hernandez-Morales et al., 2018), little is known regarding their efficacy as therapeutic agents *in vivo*. In our previous study, a capsule depolymerase Dpo48 was identified and characterized (Liu et al., 2019). *In vitro* experiments showed that the enzyme-treated *A. baumannii* were significantly decreased in the presence of serum complement. Here, we explored its potential as a therapeutic agent to control systemic infections induced by extensively drug-resistant *A. baumannii* using *Galleria mellonella* and mice infection models. Hemolysis assay on human red blood cells and cytotoxicity assays on human lung, liver and kidney cell lines were performed to confirm the safety of this enzyme for human application.

## Materials and Methods

### *A. baumannii* Strain and the Capsule Depolymerase Dpo48

The extensively drug-resistant *A. baumannii* AB1610 was isolated from sputum samples from patients with severe pneumonia in the intensive care unit at 307th Hospital of Chinese People’s Liberation Army, Beijing, China. This *A. baumannii* strain was identified by 16S ribosomal rRNA gene sequencing, and its antibiotic resistance profile was shown in our previous report (Liu et al., 2019).

Based on the annotation results of the phage IME200 sequence (GenBank accession number KT804908.2), the open reading frame 48 (GenBank accession number ALJ97635) was predicted to encode the capsule depolymerase Dpo48 with a predicted molecular weight of 75.141 kDa. The expression, purification and identification of this depolymerase, Dpo48, were described in detail previously (Liu et al., 2019). Briefly, the coding sequence of Dpo48 was cloned into the prokaryotic expression vector pET28a (Novagen, Madison, WI, United States), and the recombinant plasmid was transfected into the *Escherichia coli* BL21 cells. The protein was expressed under 0.1 mM isopropyl β-D-1-thiogalactopyranoside incubated at 16°C overnight. Then the sample was loaded into a Ni-nitrilotriacetic acid column (Sangon Biotech, Shanghai, China) and eluted with five volumes of imidazole-containing buffer (50 mM NaH_2_PO_4_, 300 mM NaCl, 250 mM imidazole, pH 8.0) via a step gradient to collect the purified Dpo48. The collected depolymerase was dialyzed with phosphate buffered saline (PBS) at 4°C overnight using a dialysis bag of 8–14 kDa molecular-mass-cutoff membrane (Viskase, Willowbrook, IL, United States). The concentration of Dpo48 was determined to be 0.5 mg/mL by the Bradford Protein Assay Kit (Thermo Fisher Scientific, MA, United States). The protein was stored at -80°C before use.

### *Galleria mellonella* Infection Model

The treatment efficacy of Dpo48 *in vivo* was first tested in a *G. mellonella* infection model which is well established to assess novel therapeutics for bacterial pathogens (Peleg et al., 2009; Jacobs et al., 2014). The *G. mellonella* weighing about 250∼300 mg (Tianjin Huiyude Biotech Company, Tianjin, China) were used within 7 days of shipment from the vendor and kept in the dark at 21°C before infection. The 100% minimal lethal dose (MLD_100_) of *G. mellonella* was conducted as described previously (Peleg et al., 2009; Jacobs et al., 2014) with slight modifications. Briefly, a single clone of *A. baumannii* AB1610 was cultured in Luria-Bertani (LB; OXOID, Hampshire, United Kingdom) broth medium overnight at 37°C. Then, the overnight culture was washed three times with PBS. *G. mellonella* were infected by injecting the bacteria inoculum at a dose of 10^7^, 10^6^, 10^5^, and 10^4^ colony-forming units (CFU) in 10 μL PBS into the last left proleg using a 10 μL Hamilton syringe (Hamilton, Reno, NV, United States). The *G. mellonella* were then incubated at 37°C and observed at 12 h intervals over 3 days. The *G. mellonella* which did not respond to physical stimuli were considered dead. Each group included 10 *G. mellonella* with individual experiments repeated three times (*n* = 30). The MLD_100_ of *G. mellonella* determined to be 10^6^ CFU by the Reed and Muench method (Supplementary Table S1) and used for the antivirulent experiment.

For the antivirulent experiment, *G. mellonella* were randomly divided into six groups. The positive control group was injected with 10^6^ CFU bacteria (*n* = 30); one treatment group was injected with bacteria, which was pretreated with Dpo48 (final concentration, 50 μg/mL) for 1 h at 37°C and washed with PBS before final injection (*n* = 30); another treatment group was injected with 10^6^ CFU bacteria and 5 μg Dpo48 in 10 μL PBS was administered within 5 min after bacterial infection (*n* = 30); the non-injected *G. mellonella* and *G. mellonella* injected with 10 μL PBS or Dpo48 as the negative control groups.

### Assessment of Acute Toxicity of Dpo48 to Mice

Normal BALB/c female mice (19∼21 g, specificpathogen-free) were purchased from SPF Biotechnology Co., Ltd. (Beijing, China). All mice were housed in regulation boxes and given free access to food and water. All animal studies were performed according to the institutional guidelines for animal welfare, and approved by the Peking University People’s Hospital Institutional Animal Care and Use Committee. Mice were sacrificed by CO_2_ asphyxiation at the end of the experimental period in accordance with institutional guidelines.

To study the potential acute toxicity of Dpo48 *in vivo*, 50 μg (in 200 μL PBS) purified enzyme or 200 μL PBS was administered intraperitoneally (i.p.) to normal mice. Six mice from each group were sacrificed for blood biochemical analyses and histopathological observation after 24 h inoculation. The survival rate, body weight and abnormal behavior of other mice (*n* = 6) were recorded daily for 7 days.

### Blood Biochemical Assays and Histopathological Observation

Blood samples of mice were centrifuged at 2000 rpm for 15 min to obtain serum, and some biochemical parameters of mice were determined by a Hitachi 7080 biochemical analyzer (Hitachi, Japan). Serum levels of total bilirubin (TBIL), aspartate aminotransferase (AST), alanine aminotransferase (ALT), and alkaline phosphatase (ALP) were measured to evaluate the liver function of mice. Blood creatinine (Cr) and urea nitrogen (BUN) levels were determined for the assessment of nephrotoxicity. Cell membrane injury and tissue damage were evaluated based on the measurements of lactate dehydrogenase (LDH).

For histopathological observation, liver, spleen, lung and kidney tissues were immediately fixed in 10% neutral buffered formalin, and subsequently embedded by paraffin. Tissues were cut into 4∼6 μm sections, stained with hematoxylin-eosin (HE; Solarbio, Beijing, China), and examined under light microscope (Ci-S, Nikon, Tokyo, Japan) for neutrophil infiltration and inflammatory changes.

### Spreading of *A. baumannii* in Mice Organs

In order to determine the spreading of *A. baumannii* in mice organs, the MLD_100_ of mice was first assayed according to the method of assessing the MLD_100_ of *G. mellonella*. In brief, *A. baumannii* AB1610 was administered i.p. to each group of mice (*n* = 6) at a dose of 10^8^, 5 × 10^7^, 2 × 10^7^, 10^7^, 5 × 10^6^, 2 × 10^6^, or 10^6^ CFU in 200 μL PBS, and observed for 7 days. A dose of 10^7^ CFU of *A. baumannii* was determined as the MLD_100_ of mice by the Reed and Muench method (Supplementary Table S2). Next, three mice were injected i.p. with a dose of 10^7^ CFU of *A. baumannii*, and euthanized 2 h postinoculation. Blood samples were obtained by the aorta abdominalis. Then, the liver, spleen, lung and kidney were aseptically removed from mice, weighed and homogenized in PBS. Serially diluted blood and homogenized tissues were plated onto brain heart infusion (BHI; BD, MD, United States) agar and incubated at 37°C for 24 h to determine bacterial counts. The amounts of bacteria in blood and organs were represented as CFU/mL and CFU per gram (g) of tissue, respectively.

### Treatment of Dpo48 on Normal or Immunocompromised Mice With Peritoneal Sepsis

A total of 22 mice were inoculated i.p. with a dose of 10^7^ CFU of *A. baumannii* and randomly divided into two groups: treated with 50 μg Dpo48 in 200 μL PBS or 200 μL PBS i.p. 2 h after bacterial challenged. Three mice of each group were euthanized 4 h posttreatment, and blood, liver, spleen, lung, and kidney samples were obtained for bacterial counting. The survival rate of other challenged mice (*n* = 8) were recorded for 7 days.

Immunocompromised mice model was developed by an i.p. injection of cyclophosphamide (CTX) with a single dose of 300 mg/kg (Thermo Fisher Scientific) in 200 μL PBS 3 days before bacterial challenge (Wang et al., 2016). The white blood cell numbers were counted at 0 and 3 days postinjection. The MLD_100_ of immunocompromised mice (Supplementary Table S3) and the efficacy of the Dpo48 therapy on the bacterial challenged immunocompromised mice were determined using the methods described above.

### Hemolysis Assay

The effect of Dpo48 on the hemolysis of human red blood cells was performed using previously described methods (Wang et al., 2015; Oliveira et al., 2018) with minor modifications. The human blood sample from a healthy donor was centrifuged at 1000 rpm for 10 min to remove the serum. The red blood cell pellets were washed with PBS (pH = 7.4) at least three times and then diluted to a concentration of 5% (v/v) with PBS. The Dpo48 (final concentration, 7.5 μg/mL) was added to the red blood cells and incubated at 37°C for 1 h with gentle shaking at 60 rpm, followed by centrifugation at 1000 rpm for 10 min. A volume of 100 μL supernatant was transferred to a 96-well microplate and topped up with another 100 μL of PBS to get a final volume of 200 μL. The erythrocytes in PBS and 0.1% Triton X-100 were recorded as negative and positive controls, respectively. The hemoglobin in supernatant was determined by measuring absorbance at 540 nm using a Synergy HT Multi-Detection Microplate Reader (BioTek, VT, United States). The experiments were performed in triplicate.

### Cytotoxicity Assays of Dpo48 on Human Cell Lines

To evaluate the acute toxicity of Dpo48 toward human *in vitro*, HuH-7 (human liver cancer), A549 (human lung carcinoma) and 293T (human embryonic kidney) cell lines were obtained from ATCC (VA, United States) and used in this research. All cells were cultured in Dulbecco’s Modified Eagle’s Medium (DMEM; Gibco, NY, United States) supplemented with 10% fetal bovine serum (FBS; Gibco) under a humidified atmosphere of 5% CO_2_ at 37°C, and sub-cultured every 2 days. The cytotoxicity of Dpo48 was determined by MTT assay according to previously methods with minor modifications (Oliveira et al., 2018). Briefly, cells were added into 96-well microtiter plates (5 × 10^3^ cells/well) and incubated at 37°C for 24 h. The culture medium was removed, and cells were washed with PBS (pH = 7.4). Then, a final concentration of 7.5 μg/mL Dpo48 or PBS was added to cells and incubated at 37°C for 24 h. The toxicity of Dpo48 toward cells was conducted using the MTT Cell Proliferation and Cytotoxicity Assay Kit (Solarbio). Quantification of soluble formazan produced by cellular reduction of MTT was measured at 540 nm using a Synergy HT Multi-Detection Microplate Reader. The experiments were performed in triplicate.

### Statistical Analysis

All experimental data are expressed as mean ± one standard deviation (SD). The independent Student’s *t* test was utilized to compare two groups, and the one-way analysis of variance (ANOVA) was used to compare multiple groups. Survival rate of *G. mellonella* or mice was calculated by Kaplan-Meier survival analysis with a log-rank test. Result analysis were performed and plotted in Prism 7 (GraphPad software, CA, United States), with *P* < 0.05 considered statistically significant.

## Results

### Antivirulent Efficacy of Dpo48 in the *G. mellonella* Infection Model

**FIGURE 1:**
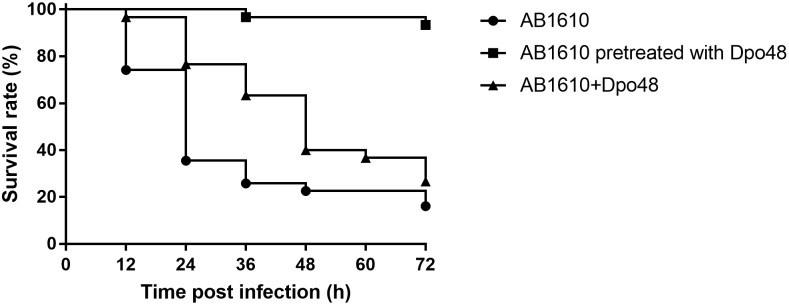
Antivirulent efficacy of Dpo48 in the *Galleria mellonella* infection model. *G. mellonella* were inoculated with *Acinetobacter baumannii* AB1610 (10^6^ CFU), or Dpo48-pretreated bacteria, or 5 μg enzyme within 5 min postinfection. Mortality of *G. mellonella* was recorded at 12 h intervals for 3 days. Kaplan-Meier survival analysis with a log-rank test was conducted. Survival rates of the enzyme-pretreated bacteria group (*n* = 30, *P* < 0.0001) and Dpo48 treatment group (*n* = 30, *P* < 0.05) were significantly higher than that of untreated group.

To assess the antivirulent efficacy of Dpo48, *G. mellonella* were injected with bacteria, Dpo48-pretreated bacteria, or Dpo48 within 5 min after bacterial challenge. As shown in Figure 1, approximately 65% of the *G. mellonella* died within 24 h after inoculated with 10^6^ CFU of *A. baumannii* AB1610. On the contrary, the survival rate of Dpo48-pretreated bacteria group and Dpo48 treatment group was 100 and 76%, respectively. The mortality rate of untreated *G. mellonella* increased to 84% after 3 days. Administration of Dpo48 after bacterial challenged could only improve the survival rate of *G. mellonella* slightly to 26% (*P* < 0.05, log-rank test), whereas more than 93% of the *G. mellonella* challenged with enzyme-pretreated bacteria (*P* < 0.0001, log-rank test) survived after 3 days. *G. mellonella* received no injection, injected with PBS or Dpo48 showed a 100% survival rate.

### Acute Toxicity Study of Dpo48 to Mice

**FIGURE 2:**
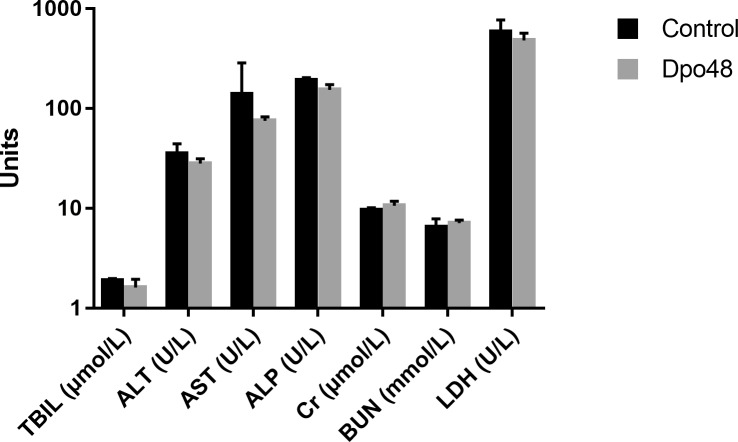
Blood biochemical assays of mice. Normal mice were administered i.p. with Dpo48 (50 μg) or PBS, and sacrificed for blood biochemical analyses at 24 h postinjection. Serum levels of total bilirubin (TBIL), aspartate aminotransferase (AST), alanine aminotransferase (ALT), alkaline phosphatase (ALP), blood creatinine (Cr), urea nitrogen (BUN) and lactate dehydrogenase (LDH) were determined. Data are presented as means ± *SD* (*n* = 6), and the statistical analysis was assayed by the independent Student’s *t* test (*P* > 0.05).

**FIGURE 3:**
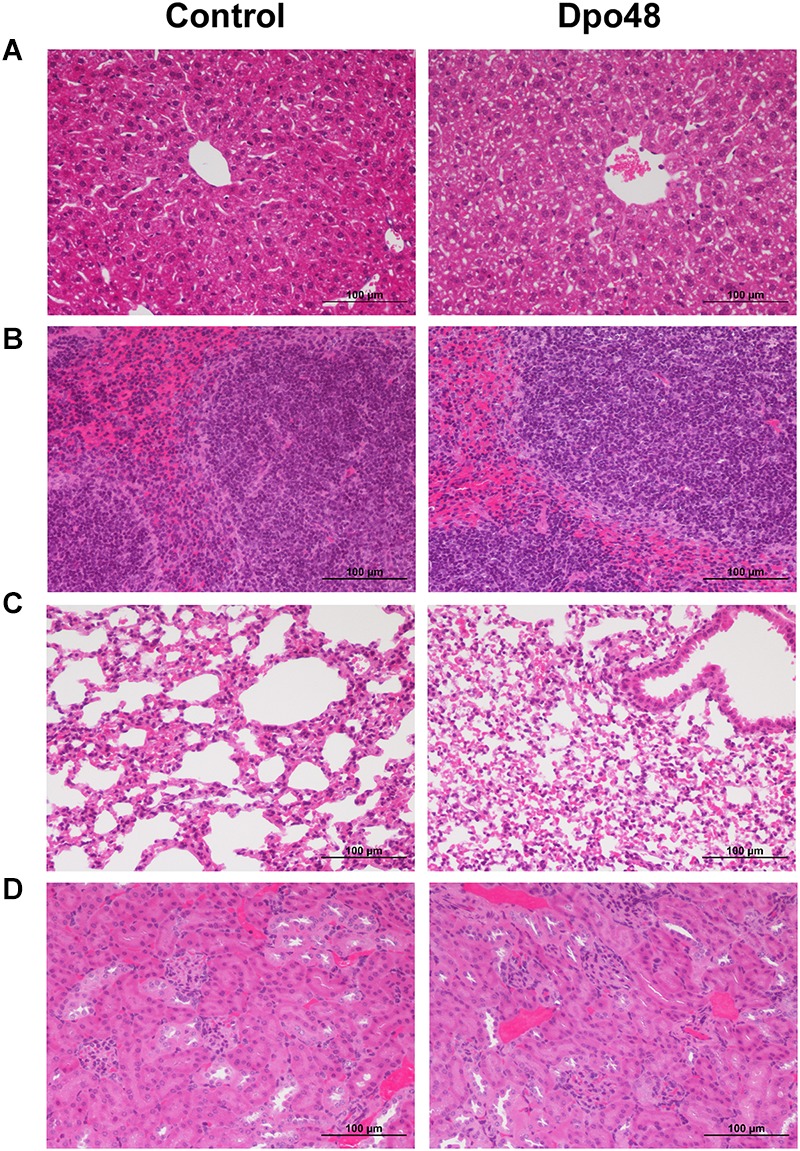
Histopathology of mice tissues. Normal mice were injected i.p. with Dpo48 (50 μg) or PBS, and sacrificed for histopathological observation at 24 h postinjection. Tissues of liver **(A)**, spleen **(B)**, lung **(C)** and kidney **(D)** were stained with hematoxylin-eosin, and observed and recorded under light microscope (200×). There were no significantly histopathological changes in tissues between the control and Dpo48-treated mice.

In order to evaluate the acute toxicity of Dpo48 to mice, the blood biochemical analyses of normal mice inoculated with PBS or enzyme were conducted, and the histopathological changes of mice tissues were examined. As depicted in Figure 2, there were no significant differences in serum levels of TBIL, AST, ALT, ALP, Cr, BUN, and LDH between the two groups after 24 h inoculation. Furthermore, no significantly histopathological changes were observed in tissues of liver, spleen, lung and kidney between the control and Dpo48-treated mice (Figure 3). All mice survived and appeared healthy without any abnormal behavior and obvious differences in body weight gain between the two groups during the 7-day observation period (data not shown). Therefore, the injected Dpo48 dose, 50 μg, caused no or little acute toxicity to mice.

### Efficacies of Dpo48 Therapy on Mice Peritoneal Sepsis

**FIGURE 4:**
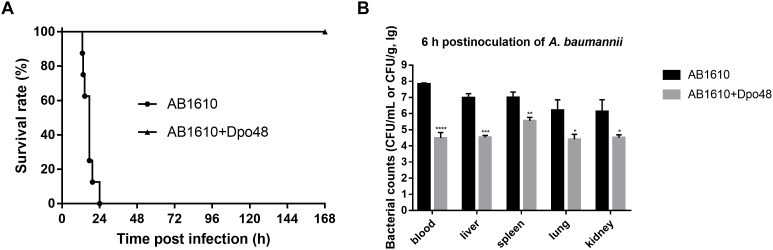
Dpo48 rescues mice from peritoneal sepsis induced by *A. baumannii*. Normal mice were inoculated i.p. with *A. baumannii* AB1610 (10^7^ CFU), and injected with Dpo48 (50 μg) or PBS at 2 h postinfection. **(A)** Mortality of mice was observed for 7 days, and comparison of the survival rates between the two groups was determined by Kaplan-Meier survival analysis with a log-rank test (*n* = 8, *P* < 0.0001). **(B)** Mice in each group were euthanized at 6 h postinoculation of *A. baumannii*, and the blood samples, liver, spleen, lung, and kidney were obtained and plated onto BHI agar for bacterial counting. The bacterial numbers are indicated as means ±*SD* (*n* = 3), and the data analysis was conducted by the independent Student’s *t* test (^∗^*P* < 0.05, ^∗∗^*P* < 0.01, ^∗∗∗^*P* < 0.001, ^∗∗∗∗^*P* < 0.0001).

After 2 h inoculation of *A. baumannii*, the average bacterial counts in liver, spleen, lung, kidney, and blood samples of mice reached 1.1 × 10^6^, 1.2 × 10^6^, 3.4 × 10^4^, 7.2 × 10^4^ CFU/g, and 1.5 × 10^6^ CFU/mL (Supplementary Figure S1). Bacteria had been rapidly spread to blood and organs of mice by i.p. infection, suggesting that the *A. baumannii* infection was severe and systemic. A dose of 50 μg Dpo48 was injected into one group of mice 2 h postinoculation to evaluate the therapeutic efficacies of the enzyme. Survival rates of mice indicated that all mice injected with 10^7^ CFU of *A. baumannii* died within 24 h postinoculation without treatment, whereas the infected mice treated with Dpo48 led to a 100% survival rate and appeared healthy through the 7-day observation period (Figure 4A). In other words, Dpo48 could effectively treat mice peritoneal sepsis caused by *A. baumannii* (*P* < 0.0001, log-rank test). Besides, the bacterial counts in blood and tissue samples of mice treated with Dpo48 were significantly decreased compared with that of the untreated mice 6 h postinoculation (Figure 4B).

### Efficacies of Dpo48 Therapy on Immunocompromised Mice With Peritoneal Sepsis

**FIGURE 5:**
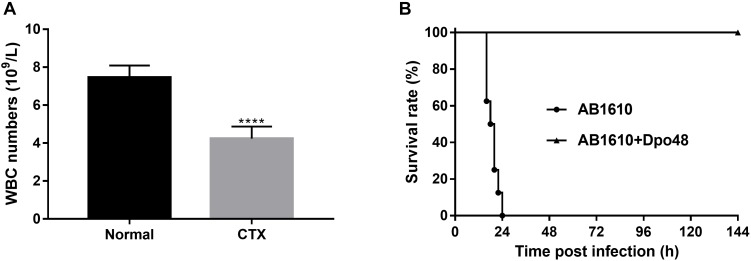
Treatment application of Dpo48 on immunocompromised mice with peritoneal sepsis caused by *A. baumannii*. **(A)** Cyclophosphamide (300 mg/kg) was injected i.p. into normal mice. The white blood cell numbers of mice were determined at 0 and 3 days postinjection. The cell counts are shown as means ±*SD* (*n* = 6), and the comparison of two groups was adapted to the Student’s *t* test (^∗∗∗∗^*P* < 0.0001). **(B)** The immunocompromised mice were injected i.p. with *A. baumannii* AB1610 (10^7^ cfu), and administered with Dpo48 (50 μg) or PBS at 2 h postinfection. Mice were observed for 7 days for mortality and the survival rate comparison was calculated by Kaplan-Meier survival analysis with a log-rank test (*n* = 8, *P* < 0.0001).

Cyclophosphamide is an alkylating agent that interferes with DNA replication, which is often used as an immunosuppressive agent in rodent animal model development. It could inhibit mouse immune response or deteriorate its healthy status, which mimics the situation in clinical setting that *A. baumannii* often target immunocompromised patients. As depicted in Figure 5A, the white blood cell counts of the CTX-treated mice were significantly decreased 3 day postinjection (*P* < 0.0001). The immunocompromised mice also appeared with symptoms of slow movement, crowding together, eyes shut and ruffled fur. After injection with 10^7^ CFU *A. baumannii*, all untreated immunocompromised mice died within 24 h infection. On the contrary, the survival rate of mice treated with Dpo48 at 2 h postinfection was 100% within the 7-day observation period (Figure 5B), with the mice gradually becoming more active. The results suggested that the immunocompromised mice were able to defense effectively against the enzyme-treated bacteria (*P* < 0.0001, log-rank test).

### The Hemolysis and Toxicity of Dpo48 Toward Human Cells

**FIGURE 6:**
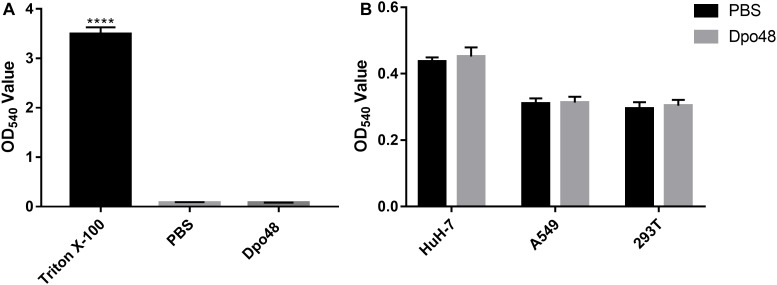
Hemolysis and toxicity effect of Dpo48 on mammalian cells. **(A)** The Dpo48 (7.5 μg/mL) was added to a concentration of 5% (v/v) red blood cells and incubated at 37°C for 1 h with gentle shaking at 60 rpm, and the erythrocytes in PBS and 0.1% Triton X-100 were recorded as negative and positive controls, respectively. The hemoglobin of supernatant was determined at 540 nm. Data are represented as means ±*SD* (*n* = 6), and the one-way ANOVA was used to statistical analysis (^∗∗∗∗^*P* < 0.0001). **(B)** HuH-7 (human liver cancer), A549 (human lung carcinoma), and 293T (human embryonic kidney) cell lines were seeded into 96-well microtiter plates (5 × 10^3^ cells/well) and incubated at 37°C for 24 h. The Dpo48 (7.5 μg/mL) or PBS was added to cells and incubated at 37°C for 24 h. The quantification of soluble formazan produced by cellular reduction of MTT was measured at 540 nm. The values are expressed as means ±*SD* (*n* = 6), and the independent Student’s *t* test was adopted in two groups comparison (*P* > 0.05).

The toxic effects of Dpo48 on human red blood cells and liver cancer, lung carcinoma and embryonic kidney cell lines were assessed *in vitro* to confirm the potential application and safety of the depolymerase to human. As shown in Figure 6A, Dpo48 displayed no hemolytic activity to erythrocytes based on the determination of hemoglobin in the supernatant. The enzyme also showed no toxic effects toward other human cells according to the quantification of soluble formazan produced by cellular reduction in the MTT assay (Figure 6B).

## Discussion

Antibiotics are important in curing minor or life threatening infections caused by bacteria. However, most clinical bacterial strains have shown to be resistant to at least three classes of antibiotics, presenting a significant global medical challenge for treatment of infections caused by superbugs. Alarmingly, there is no new antibiotics have reached the advanced development stages for infections caused by multidrug-resistant gram-negative bacteria (World Health Organization, 2017). Phage, as natural host of bacteria, have been refocused by many researchers on a global scale (Wright et al., 2009; Fish et al., 2016; Leitner et al., 2017; Schooley et al., 2017) and demonstrated as an effective alternative to antibiotics in combating drug-resistant bacteria. However, the current regulations bear significant challenges to allow full exploration of the benefits of this new therapeutic agent. Contrarily, phage-encoded endolysins and depolymerases, which have been identified as new class of antimicrobials, can follow the development of protein-based pharmaceutics, holding a greater potential to the market.

For most gram-negative bacteria, capsule polysaccharides are major determinants for promoting host virulence, and protection from phagocytosis and antimicrobials (Roach and Donovan, 2015). In our previous study, we have demonstrated the ability of Dpo48 to effectively degrade bacterial capsules and subsequently exposing the inner bacteria for immune attack *in vitro*. It is important to further evaluate the anti-virulence efficacy of this enzyme *in vivo*. Typically, murine or other rodent models were used to assess the toxicity and efficacy of new antimicrobial agents *in vivo*. However, this experimentation is not only costly and time consuming, but also requires full ethical consideration (Desbois and Coote, 2012). Hence, the antivirulent efficacy of Dpo48 in this research was first tested in a *G. mellonella* infection model because of its demonstrated suitability in assessing the efficacy of novel therapeutics for *A. baumannii* infections immediately prior to mammalian studies (Peleg et al., 2009; Jacobs et al., 2014). The better survival rate (10% higher, *P* < 0.05) of infected *G. mellonella* treated with Dpo48 compared with the untreated group confirmed that the enzyme could be an antivirulent agent to control *A. baumannii* infections *in vivo*. The results were in line with findings by Grazyna Majkowska-Skrobek (Majkowska-Skrobek et al., 2016). Although the treatment efficacy was not ideal, the much higher survival rate of the *G. mellonella* (93%) injected with enzyme-pretreated bacteria suggested that the loss of surficial capsules might cause the decrease the virulence of *A. baumannii*. Therefore, a part of the non-encapsulated *A. baumannii* might be cleared up by the innate immune system of *G. mellonella*, and the counts of remaining bacteria might not enough cause the death of *G. mellonella* despite the bacteria would restore the capsule during the propagation.

If depolymerases were considered to be used as therapeutic agents *in vivo*, the evaluation of their acute toxicity to mammalian tissues or cells was important. Mice injected with Dpo48 showed normal serum levels of TBIL, AST, ALT, ALP, Cr, BUN, and LDH after 24 h (Figure 2). Moreover, no significant histopathology changes were observed in mice tissues of liver, spleen, lung, and kidney (Figure 3), in agreement with other findings (Pan et al., 2015). The enzyme was also founded to be a non-hemolytic and non-toxic agent toward human cells (Figure 6A,B). However, the histidine tag and endotoxins in the Dpo48 preparation will need to be removed or reduced to lower levels before used in human trials.

Our previous study (Liu et al., 2019) indicated that a small amount of Dpo48 (10 μg/mL) could effectively strip the capsules of overnight *A. baumannii* with which subsequently killed by serum complement *in vitro*. Although previous research has proved that depolymerases could prevent rat or mice death from lethal bacteraemia, studies were focused on controlling systemic infections caused by *E. coli* and *Klebsiella pneumonia* infections (Mushtaq et al., 2004, 2005; Zelmer et al., 2010; Lin et al., 2014, 2017; Pan et al., 2015; Hsieh et al., 2017). This is the first study to evaluate the efficacy of a depolymerase, Dpo48, in treating *A. baumannii* infections *in vivo*. In addition, most previous studies demonstrated the efficacy of depolymerases against gram-negative bacteria were performed with co-administration of depolymerases and pathogenic bacteria at the same time (Mushtaq et al., 2005; Lin et al., 2014; Hsieh et al., 2017). In other words, the treatment could start before the pathogenic bacteria spread in all animal organs. This means these therapeutic methods might not reflect the clinical situation that treatments only take place after spreading of bacteria in systemic infections. To better representing the actual clinical practice, the present study was performed with the Dpo48 treatment initiated when the *A. baumannii* infection in mice was severe and systemic.

The 100% survival rates of the infected normal and immunocompromised mice revealed that Dpo48 could effectively treat peritoneal sepsis induced by *A. baumannii*. Comparing with the normal mice, the health status of immunocompromised mice was deteriorated. However, they were still capable of clearing the enzyme-degraded *A. baumannii*, because the level of serum complement was not reduced by cyclophosphamide (Li et al., 1987). This observation suggested that the complement-dependent mechanism is responsible for the clearance of enzyme-treated bacteria and protection for infection (Lin et al., 2014; Majkowska-Skrobek et al., 2018). As immunocompromised patients are at higher risk of developing *A. baumannii* infections, the exceptional survival rate of Dpo48 treated immunocompromised mice demonstrated clearly the potential of this enzyme against *A. baumannii* infections in clinical setting. The Dpo48 had better treatment efficacy in mice than that in *G. mellonella* could be due to the simpler innate immune system of insects. The insects only depend on germline-encoded factors for recognition and clearance pathogens (Wojda, 2017). Therefore, the insect models cannot replace the mammalian infection models completely. However, they can provide a rapid and economical approach to observe the effects of a new antimicrobial compound prior to further testing in a mammalian infection model (Desbois and Coote, 2012).

In conclusion, the safety and efficacy results suggested that the phage-encoded depolymerase, Dpo48, have a great potential as a therapeutic agent to prevent and control severe infections caused by *A. baumannii* in *G. mellonella* and mice models. However, the applicability of depolymerases against human infections still needs to be supported by further clinical trials.

## Author Contributions

YL conceived the project, designed the experiments, analyzed the data, and wrote the manuscript. SL helped to revise and edit the manuscript. YL, YG, LZ, LM, and CW performed the biological experiments. YL, NJ, PL, and YQ prepared all figures and tables. ZM, CB, and ZG helped to conceive the project and design the experiments, and contributed to reagents or materials. All authors reviewed the manuscript.

## Conflict of Interest Statement

The authors declare that the research was conducted in the absence of any commercial or financial relationships that could be construed as a potential conflict of interest.

## Supplementary Material

FIGURE S1 Spreading of *A. baumannii* in mice blood and organs. Normal mice were injected i.p. into a dose of 10^7^ CFU of *A. baumannii* AB1610 and euthanized at 2 h postinoculation. The bacterial counts of mice blood, liver, spleen, lung, and kidney were assayed, and data are expressed as means ±*SD* (*n* = 3).

TABLE S1 Determination the MLD_100_ of *Galleria mellonella* by using the Reed and Muench method.

TABLE S2 Determination the MLD_100_ of Mice by using the Reed and Muench method.

TABLE S3 Determination the MLD_100_ of Immunocompromised Mice by using the Reed and Muench method.
