# Corrigendum: The Capsule Depolymerase Dpo48 Rescues *Galleria mellonella* and Mice From *Acinetobacter baumannii* Systemic Infections

**DOI:** 10.3389/fmicb.2019.00942

**Published:** 2019-04-30

**Authors:** Yannan Liu, Sharon Shui Yee Leung, Yatao Guo, Lili Zhao, Ning Jiang, Liyuan Mi, Puyuan Li, Can Wang, Yanhong Qin, Zhiqiang Mi, Changqing Bai, Zhancheng Gao

**Affiliations:** ^1^Department of Respiratory and Critical Care Medicine, Peking University People's Hospital, Beijing, China; ^2^School of Pharmacy, The Chinese University of Hong Kong, Shatin, Hong Kong; ^3^State Key Laboratory of Pathogen and Biosecurity, Beijing Institute of Microbiology and Epidemiology, Beijing, China; ^4^Department of Respiratory and Critical Care Medicine, 307th Hospital of Chinese People's Liberation Army, Beijing, China

**Keywords:** *Acinetobacter baumannii*, capsule depolymerase, *Galleria mellonella* model, mouse model, systemic infection, antivirulence

In the published article, there was an error in the affiliation of Yannan Liu. The author is not connected to the second affiliation, 307th Hospital of Chinese People's Liberation Army. The authors apologize for this error and state that this does not change the scientific conclusions of the article in any way. The original article has been updated.

