# Supplementary figures and images for: The Capsule Depolymerase Dpo48 Rescues Galleria mellonella and Mice From Acinetobacter baumannii Systemic Infections
Source: Front Microbiol. 2019 Mar 18;10:545. doi: 10.3389/fmicb.2019.00545 (PMC6431613; doi:10.3389/fmicb.2019.00545)

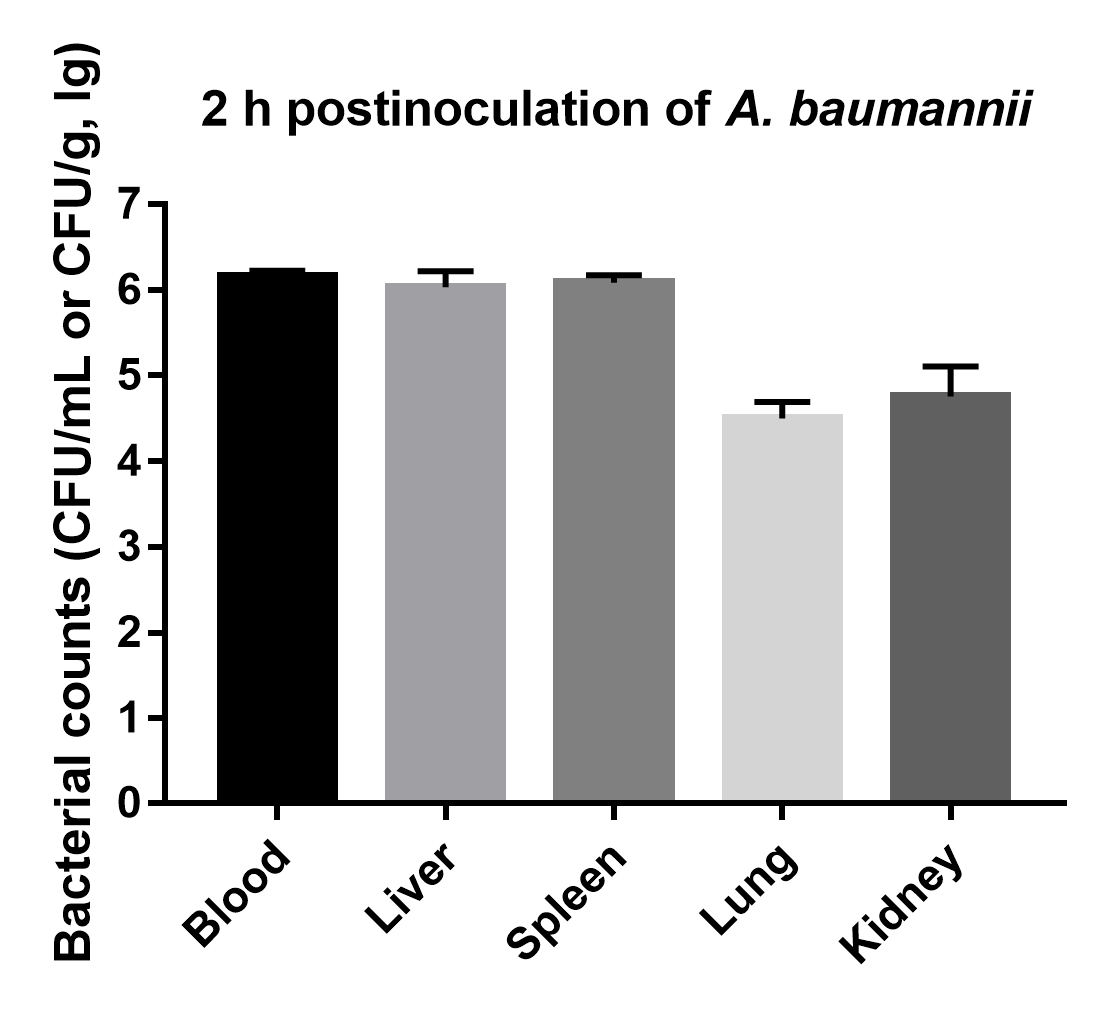

Supplement: FIGURE S1 — Spreading of A. baumannii in mice blood and organs. Normal mice were injected i.p. into a dose of 107 CFU of A. baumannii AB1610 and euthanized at 2 h postinoculation. The bacterial counts of mice blood, liver, spleen, lung, and kidney were assayed, and data are expressed as means ±SD (n = 3). [file Image_1.TIF]
