# Supplementary material for: The Capsule Depolymerase Dpo48 Rescues Galleria mellonella and Mice From Acinetobacter baumannii Systemic Infections
Source: Front Microbiol. 2019 Mar 18;10:545. doi: 10.3389/fmicb.2019.00545 (PMC6431613; doi:10.3389/fmicb.2019.00545)
Supplement: TABLE S1 — Determination the MLD100 of Galleria mellonella by using the Reed and Muench method. [file Table_1.DOCX]

**Supplementary Table 1** Determination the MLD_100_ of *Galleria mellonella* by using the Reed and Muench method.

| Bacteria (CFU) | Died | Survived | Accumulated values | | Mortality | |
| --- | --- | --- | --- | --- | --- | --- |
|  |  |  | Died | Survived | Ratio | Ratio (100 %) |
| 10^7^ | 30 | 0 | 62 | 0 | 62/62 | 100 |
| 10^6^ | 29 | 1 | 32 | 1 | 32/33 | 97 |
| 10^5^ | 3 | 27 | 3 | 28 | 3/31 | 9.7 |
| 10^4^ | 0 | 30 | 0 | 58 | 0/58 | 0 |
