# Supplementary material for: The Capsule Depolymerase Dpo48 Rescues Galleria mellonella and Mice From Acinetobacter baumannii Systemic Infections
Source: Front Microbiol. 2019 Mar 18;10:545. doi: 10.3389/fmicb.2019.00545 (PMC6431613; doi:10.3389/fmicb.2019.00545)
Supplement: TABLE S2 — Determination the MLD100 of Mice by using the Reed and Muench method. [file Table_2.DOC]

**Supplementary Table 2** Determination the MLD_100_ of Mice by using the Reed and Muench method.

| Bacteria (CFU) | Died | Survived | Accumulated values | | Mortality | |
| --- | --- | --- | --- | --- | --- | --- |
|  |  |  | Died | Survived | Ratio | Ratio (100 %) |
| 10^8^ | 6 | 0 | 31 | 0 | 31/31 | 100 |
| 5×10^7^ | 6 | 0 | 25 | 0 | 25/25 | 100 |
| 2×10^7^ | 6 | 0 | 19 | 0 | 19/19 | 100 |
| 10^7^ | 6 | 0 | 13 | 0 | 13/13 | 100 |
| 5×10^6^ | 4 | 2 | 7 | 2 | 7/9 | 78 |
| 2×10^6^ | 3 | 3 | 3 | 5 | 3/8 | 38 |
| 10^6^ | 0 | 6 | 0 | 11 | 0/11 | 0 |
