# Supplementary material for: The Capsule Depolymerase Dpo48 Rescues Galleria mellonella and Mice From Acinetobacter baumannii Systemic Infections
Source: Front Microbiol. 2019 Mar 18;10:545. doi: 10.3389/fmicb.2019.00545 (PMC6431613; doi:10.3389/fmicb.2019.00545)
Supplement: TABLE S3 — Determination the MLD100 of Immunocompromised Mice by using the Reed and Muench method. [file Table_3.DOC]

**Supplementary Table 3** Determination the MLD_100_ of Immunocompromised Mice by using the Reed and Muench method.

| Bacteria (CFU) | Died | Survived | Accumulated values | | Mortality | |
| --- | --- | --- | --- | --- | --- | --- |
|  |  |  | Died | Survived | Ratio | Ratio (100 %) |
| 10^7^ | 6 | 0 | 17 | 0 | 17/17 | 100 |
| 5×10^6^ | 6 | 0 | 11 | 0 | 11/11 | 100 |
| 2×10^6^ | 4 | 2 | 5 | 2 | 5/7 | 71 |
| 10^6^ | 1 | 5 | 1 | 7 | 1/8 | 13 |
| 5×10^5^ | 0 | 6 | 0 | 13 | 0/13 | 0 |
